# Importance of pediatric rheumatologists and transitional care for juvenile idiopathic arthritis-associated uveitis: a retrospective series of 9 cases

**DOI:** 10.1186/s12969-020-0419-1

**Published:** 2020-03-23

**Authors:** Susumu Yamazaki, Asami Shimbo, Yuko Akutsu, Hiroshi Takase, Tomohiro Morio, Masaaki Mori

**Affiliations:** 1 Department of Lifetime Clinical Immunology, Graduate School of Medical and Dental Sciences, Tokyo Medical and Dental University, 1-5-45, Yushima, Bunkyo-ku, Tokyo 113-8519 Japan; 2 Department of Pediatrics and Adolescent Medicine, Juntendo University Graduate School of Medicine, Tokyo, Japan; 3 Department of Pediatrics and Developmental Biology, Perinatal and Maternal Medicine, Graduate School of Medical and Dental Sciences, Tokyo Medical and Dental University (TMDU), Tokyo, Japan; 4 Department of Ophthalmology & Visual Science, Graduate School of Medical and Dental Sciences, Tokyo Medical and Dental University (TMDU), Tokyo, Japan

**Keywords:** Juvenile idiopathic arthritis, Transitional care, Uveitis

## Abstract

**Background:**

Juvenile idiopathic arthritis-associated uveitis (JIA-U) is a serious condition associated with the risk of blindness. However, pediatric rheumatologists rarely encounter cases of blindness, because most patients reach adulthood during the course of follow-up before blindness occurs. Here, we report the progress of 9 patients with JIA-U, including 2 patients who became blind after the transition period. We aimed to highlight the importance of the role of pediatric rheumatologists and transitional care in preventing blindness associated with JIA-U.

**Case presentation:**

We conducted a retrospective analysis of the case records of 9 JIA-U patients (1 male, 8 female; median age 16.8 years, range 5.5–19.8 years). All patients presented with oligo-juvenile idiopathic arthritis (oligo-JIA) (one presented with extended oligo-JIA); the median age of uveitis onset was 5.0 years (range 3.0–13.0 years), and the onset of uveitis preceded the onset of arthritis in 2 patients. The median disease duration was 12.5 years (range 3.5–24.7 years); 4 patients had anti-nuclear antibody (ANA) positivity (≧1:160) (all with a homogeneous and speckled-pattern subtype). All patients were negative for rheumatoid factor. Eight patients received methotrexate, 7 patients received one or more biologic drugs (etanercept, infliximab, adalimumab, and golimumab), and 6 patients required ophthalmic surgery at an early age (≦ 18 years).

Two patients developed blindness after the transition period. Medical examination by pediatric rheumatologists and use of biologics had been delayed in both patients. One patient developed depression after transition and interrupted her own treatment.

**Conclusions:**

The reason for blindness in the 2 patients was thought to be the delay in the commencement of treatment and failure to provide transitional care. Inflammation is difficult to control in JIA-U even with appropriate treatment. Pediatric rheumatologists must be informed about the risk of JIA-U blindness, especially after transition. To ensure a good prognosis, the specialized treatment with the involvement of pediatric rheumatologists is necessary early on, and consideration for transitional medicine is essential. Therefore, this report reaffirms the importance of planned transitional care that has been advocated for globally.

## Background

Uveitis is a serious extra-articular manifestation of juvenile idiopathic arthritis (JIA) associated with a risk of blindness. A long-term follow-up report of patients diagnosed with JIA-associated uveitis (JIA-U) from 1973 to 1982, prior to the availability of biologics, revealed that legal blindness developed in 5.4% of the patients (best corrected visual acuity [BCVA] ≦0.1) over 7 years [1]. However, pediatric rheumatologists rarely encounter cases of blindness due to JIA-U. Among the JIA-U cases reported in Japan, legal blindness was not noted among patients under 18 years of age [2].

Although the prevalence rate of JIA is found to be 32.6 per 100,000 /person [3], JIA-U is rarer, and it occurs in 4.7–20.5% of JIA patients, with local differences noted (6.1% reported in Japan) [2, 4–9]. Improved anti-inflammation effects with biologics has resulted in a good prognosis for JIA-U [10, 11]. However, most patients reach adulthood during the course of follow-up before blindness occurs. Notably, a longer disease duration was related to a higher risk of blindness (the incidence of legal blindness was 0.14/person-year) [12]; therefore, the risk of blindness is higher in the adult stage.

Here, we report the progress of 9 patients with JIA-U, including 2 patients who developed blindness after the transition period. In recent years, the importance of planned transitional care has been proposed globally [13, 14]. We aimed to highlight the importance of pediatric rheumatologists and transitional care in preventing blindness due to JIA-U.

### Case presentation

We conducted a retrospective analysis of the case records of 9 patients with JIA-U who received care at the Department of Pediatric Rheumatology and Department of Ophthalmology at the Tokyo Medical and Dental University from April 2015 to August 2019 (107 JIA cases). The study was conducted in adherence with the guidelines of the Declaration of Helsinki, and written informed consent was obtained from each patient and their guardians. The IRB/Ethics Committee ruled that approval was not required for this study.

**Fig. 1:**
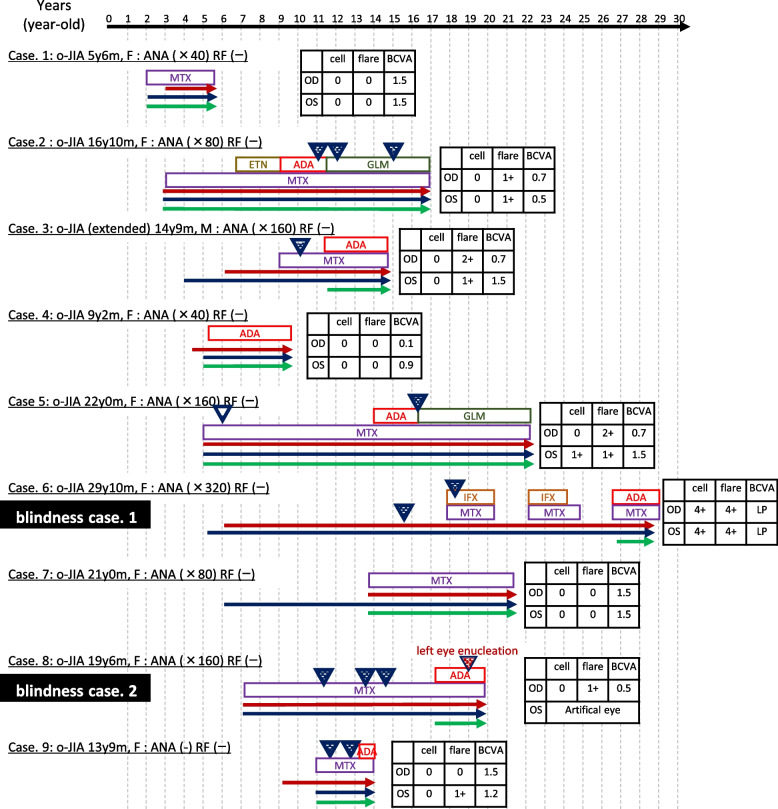
Progress and treatment of 9 cases of juvenile idiopathic arthritis-associated uveitis. Cases are presented in order of age at onset, including current age, sex, JIA category, ANA titer, RF positivity, and therapies other than topical and systemic steroids. The box shows the current or the worst status during the study periods uveitis inflammation scales based on Standard Uveitis Nomenclature criteria and current BCVA. “Cell” refers to “anterior chamber cells grade”, and “flare” refers to “anterior chamber flare grade”. Case 6 was expressed as OS cell and flare 4+ is error, correct is OS cell and flare are 0. In actually it was too cloudy due to inflammation that the number of cells could not be evaluated. L.P. of Case 6 is error, correct is no L.P., actually she could not recognize the light.” Triangles indicate surgical interventions. Red and blue lines indicate the duration of JIA-U and JIA, respectively. The green line indicates the duration of treatment by a pediatric rheumatologist. ANA: anti-nuclear antibody, RF: rheumatoid factor, BCVA: best corrected visual acuity, F: female M: male, o-JIA: oligo- juvenile idiopathic arthritis, OD: oculus dexter (right eye), OS: oculus sinister (left eye), LP (light perception) ADA: adalimumab, ETN: etanercept, GLM: golimumab, IFX: infliximab, MTX: methotrexate

Figure 1 presents the 9 cases of JIA-U in order of age at onset, including 2 cases (Cases 4 and 9) in which uveitis preceded arthritis. Risk factors associated with the development of JIA-U included sex, JIA category, age at onset, the titer of anti-nuclear antibody (ANA), human leukocyte antigen B27 (HLA-B27) positivity, and rheumatoid factor (RF) negativity [2, 15–17]. These factors were reflected in our 9 JIA-U patients as follows: median age was 16.8 years (range 5.5–19.8 years), median disease duration was 12.5 years (range 3.5–24.7 years), the sex ratio was 1:8 (male:female), all patients presented with oligo-JIA (one presented with extended oligo-JIA), median age at uveitis onset was 5.0 years (range 3.0–13.0 years), the onset of uveitis preceded arthritis in 2 patients, 4 patients showed ANA positivity (≧ 1:160) (all patients presented with the homogeneous and speckled-pattern subtype), and all patients were negative for RF. It was difficult to determine the frequency of the HLA-B27 allele, because only 1 patient was tested (the patient was HLA-B27 negative).

All patients used topical glucocorticoid eye drops, 8 patients took methotrexate, and 7 were administered biologic drugs. The biologics prescribed were etanercept, infliximab, adalimumab, or golimumab. Six patients required ophthalmic surgery for cataracts, glaucoma, or iritis at an early age (≤ 18 years). Studies show the following regarding etanercept: it may induce JIA-U [18], shows no difference from placebo [19], and is inferior to adalimumab and infliximab [20]; Etanercept is therefore no longer considered a treatment for uveitis [21–23]. However, this information was not recognized at the time, and etanercept was used for one patient (case 2).

Two patients took golimumab (cases 2, 5) after approval by the hospital ethics committee for off-label use in Japan. These 2 patients had refractory JIA-U, which was not improved by adalimumab; however, golimumab treatment resulted in improved anterior chamber inflammation after 4 weeks, observed during slit-lamp examination (based on Standard Uveitis Nomenclature criteria) [24], and an improved inflammation grade, from 3 to 1.

Details on the 2 cases of blindness are described below.

### Blindness case 1 (case 6)

A 29-year-old female patient, with no prior medical history, presented at 4 years of age with knee joint swelling and fever and was diagnosed with oligo-JIA. The arthritis disappeared within a month with nonsteroidal anti-inflammatory drug (NSAID) use. At age of 6 years, she was diagnosed with asymptomatic JIA-U; topical and systemic prednisolone use was commenced during exacerbations, but methotrexate or biologics was not prescribed (at the time as there were no indications for the use of biologics for the JIA). At age of 15 years, she complained of visual impairment, and had cataract surgery in her left eye (OS). She continued topical and systemic glucocorticoid therapy for 2 years, but her ocular inflammation was uncontrolled. At 18 years old, she was transitioned out of pediatric care and was treated as an adult patient. At that point, her BCVA was 0.02 in the right eye (OD) and 0.01 in the OS. Slit-lamp examination demonstrated 3+ anterior chamber cell/ 4+ anterior chamber flare OD, and 2+ anterior chamber cell/ 3+ anterior chamber flare OS. Soon after, cataract surgery in her OD was performed, and she was started on infliximab and methotrexate therapies. Her BCVA recovered to 0.7 (OD) / 0.08 (OS) when she was 19 years old. However, she developed depression and did not visit the outpatient clinic for 2 years. She stated that the rapid strengthening of treatment after the transition and the apathetic attitude of adult doctors kept her from returning to the hospital. She visited the hospital again at the age of 21 years with visual acuity to light perception bilaterally. Infliximab was restarted but was not effective, likely due to antibody production and the development of resistance. At the discretion of the attending physician and the patient, infliximab and methotrexate were considered invalidated; further treatment was withdrawn. At 27 years of age, she was referred to an ophthalmologist and a pediatric rheumatologist in our clinic for a second opinion. Although adalimumab (40 mg per 2 weeks) was started, no improvement was seen in the BCVA. While golimumab was tried to switch, was also no effective (It was not described in Fig. 1 because of very short-term use.) Currently, she remains legally blind but continues anti-inflammatory treatment with adalimumab and methotrexate to prevent phthisis bulbi.

### Blindness case 2 (case 8)

A 19-year-old female patient, with no previous medical history, presented at 7 years of age with right knee joint swelling and visual impairment (OS) and was diagnosed with oligo-JIA and JIA-U. The arthritis resolved within a year with NSAID, prednisolone, and methotrexate treatments, but ocular inflammation continued; thus, only methotrexate was continued thereafter. Ocular inflammation persisted, and surgeries were repeated. She underwent cataract surgery (OS) when she was 8 years old, iridotomy (OS) when she was 9 years old, and cataract surgery and peripheral iridectomy (OD) when she was 14 years old. Although she received repeated systemic prednisolone during exacerbations, she received no additional treatment with biologics. She was referred to a pediatric rheumatologist at our clinic at the age of 15 years. The patient was completely blind in OS, BCVA was 0.5 (OD), and slit-lamp examination demonstrated 4+ anterior chamber cell/ 4+ anterior chamber flare in OS and 1+ anterior chamber cell/1+ anterior chamber flare in OD. Immediately, peripheral iridectomy was performed and adalimumab (40 mg per 2 weeks) was prescribed, but the blindness did not improve. OS enucleation was performed at 17 years of age. The combination of adalimumab and methotrexate is currently being continued to control the inflammation of the OD that remains.

## Discussion

We reported a series of 9 JIA-U cases including 2 cases of blindness, with ophthalmic surgical intervention provided to 6 patients and biologic drugs administered to 7 patients. Our hospital is considered a rheumatoid specialized institute; therefore, referral bias may have played a role in increasing the concentration of rare, more complex and severe cases, with worse outcomes. JIA-U inflammation is difficult to control despite appropriate treatment. Even in the 7 patients who were not blind, 5 required biologic therapy and 4 required ophthalmic surgery in childhood. Notably, 2 patients (cases 2 and 5) needed off-label golimumab therapy. Golimumab is a fully humanized anti-TNFα monoclonal antibody that is approved for the treatment of rheumatoid arthritis, psoriatic arthritis, ankylosing spondylitis, and moderate to severe ulcerative colitis, but not for JIA-U. The American College of Rheumatology/Arthritis Foundation guidelines do not recommend using golimumab for JIA-U [22]. However, since Cordero-Coma M et al. reported that golimumab was effective in treating JIA-U in 2011 [25], there have been several case reports that suggested the effectiveness of golimumab for treatment-resistant JIA-U, without significant adverse events [25–28]. Approval by the Ethics Committee is necessary; however, treatment using off-label biologics to relieve ocular inflammation is essential in some cases, and the decision to commence treatment requires the judgement of a skilled pediatric rheumatologist. In the 7 patients without blindness, involvement of a pediatric rheumatologist from an early stage, even without joint symptoms, helped prevent blindness. Conversely, the 2 patients with blindness experienced delayed medical examination by pediatric rheumatologists, as shown by the green line in Fig. 1. This highlights the importance of early intervention by pediatric rheumatologists for a good eye prognosis.

Notably, the 2 patients developed blindness after the transition period. In case 8, blindness was thought to result from inadequate treatment in the pediatric stage, and in case 6 it was thought to be due to failure of transitional medical care.

Case 8 was not prescribed a biologic despite 3 ophthalmic surgeries. Increasing the intensity of treatment (with methotrexate and more biologics) should be considered when the intraocular inflammation has not responded to 12 weeks of topical corticosteroid treatment [22, 23]. The treatment delays in this patients might have occurred because the risk of JIA-U was not well recognized. There are few opportunities for general pediatricians to treat JIA-U due to its low prevalence and its low risk perception; hence, pediatric rheumatologists should provide education to general pediatricians regarding the dangers of JIA-U.

In case 6, treatment enhancement was delayed due to the period when the biologic was not approved, but above all, failure of transitional care was critically responsible for her blindness. She developed depression after transition, and did not visit the hospital for 2 years. The attitude of an adult doctor would not have caused the onset of depression, but it might have triggered her decision to stop medical treatment. Possible reasons for the transitioned patient’s negative perception of the attitudes of adult doctors may be due to the inability of adult doctors to interact appropriately with the patient’s parents and failure to acknowledge the independence and autonomy of the patient. A survey conducted regarding the impressions of accepting adult patients with pediatric rheumatology disorders in non-pediatric rheumatologists among councilors of the Japan College of Rheumatology revealed that 90% agreed to oversee medical care, but only 30% stated that transitioned patients could be treated without hesitation [29]. The most common reason for the hesitation to deliver adult rheumatology care for transferred JIA patients by adult rheumatologists was feeling annoyed with having to respond to the patient’s parents rather than a lack of knowledge about childhood-onset rheumatic diseases [30]. Adult doctors may underestimate the difficulty of how mental growth may be affected by the experience of a serious illness in childhood and adolescence and may consider the patient to be lacking in independence. Depression among adolescents with rheumatism was more prevalent than that among adolescents without rheumatism [31]; therefore, rheumatologists caring for patients around the time of transition should also consider providing psychological support. The 6 core elements for transition include: 1) transition policy, 2) transition tracking and monitoring, 3) transition readiness, 4) transition planning, 5) transfer of care, and 6) transfer completion [32]. In case 6, these elements could not be implemented. In particular, doctors should have prepared a period of both adult and pediatric visits during the transition to ensure “transition tracking and monitoring.”

## Conclusions

JIA-U is an intractable disease that includes inflammation that is difficult to control despite appropriate treatment. We recommend that pediatric rheumatologists provide education to general pediatricians regarding the risk of JIA-U blindness. Furthermore, pediatric rheumatologists need to provide not only drug treatments but also support during the transition to adulthood, particularly with regard to the risk of blindness after transition. For a good visual prognosis, it may be necessary to start treatment with a specialist from an early stage and continue until the transition is completed reliably, and consideration for transitional medicine is essential.

## Data Availability

Not applicable.

## Abbreviations

ADA: Adalimumab
ANA: Anti-nuclear antibody
BCVA: Best corrected visual acuity
ETN: Etanercept
GLM: Golimumab
HLA: Human leukocyte antigen
IFX: Infliximab
JIA: Juvenile idiopathic arthritis
JIA-U: Juvenile idiopathic arthritis uveitis
MTX: Methotrexate
NSAID: Nonsteroidal anti-inflammatory drugs
OD: Oculus dexter (right eye)
o-JIA: Oligo- juvenile idiopathic arthritis
OS: Oculus sinister (left eye)
PRAJ: Pediatric rheumatology association of Japan
RF: Rheumatoid factor
