# Correction: Importance of pediatric rheumatologists and transitional care for juvenile idiopathic arthritis-associated uveitis: a retrospective series of 9 cases

**DOI:** 10.1186/s12969-023-00912-w

**Published:** 2023-10-29

**Authors:** Susumu Yamazaki, Asami Shimbo, Yuko Akutsu, Hiroshi Takase, Tomohiro Morio, Masaaki Mori

**Affiliations:** 1 Department of Lifetime Clinical Immunology, Graduate School of Medical and Dental Sciences, Tokyo Medical and Dental University, 1-5-45, Yushima, Bunkyo-ku, Tokyo , 113-8519 Japan; 2 Department of Pediatrics and Adolescent Medicine, Juntendo University Graduate School of Medicine, Tokyo , Japan; 3 Department of Pediatrics and Developmental Biology, Perinatal and Maternal Medicine, Graduate School of Medical and Dental Sciences, Tokyo Medical and Dental University (TMDU), Tokyo , Japan; 4 Department of Ophthalmology & Visual Science, Graduate School of Medical and Dental Sciences, Tokyo Medical and Dental University (TMDU), Tokyo , Japan

Following publication of the original article [1], we have been notified of the following corrections:

1. The legend for Figure one, originally published as:

“Progress and treatment of 9 cases of juvenile idiopathic arthritis-associated uveitis. Cases are presented in order of age at onset, including current age, sex, JIA category, ANA titer, RF positivity, and therapies other than topical and systemic steroids. The box shows the current uveitis inflammation scales based on Standard Uveitis Nomenclature criteria and current BCVA. “Cell” refers to “anterior chamber cells grade”, and “flare” refers to “anterior chamber flare grade”. Triangles indicate surgical interventions. Red and blue lines indicate the duration of JIA-U and JIA, respectively. The green line indicates the duration of treatment by a pediatric rheumatologist. ANA: anti-nuclear antibody, RF: rheumatoid factor, BCVA: best corrected visual acuity, F: female M: male, o-JIA: oligo- juvenile idiopathic arthritis, OD: oculus dexter (right eye), OS: oculus sinister (left eye), LP (light perception) ADA: adalimumab, ETN: etanercept, GLM: golimumab, IFX: infliximab, MTX: methotrexate.”

Should read:

“Progress and treatment of 9 cases of juvenile idiopathic arthritis-associated uveitis. Cases are presented in order of age at onset, including current age, sex, JIA category, ANA titer, RF positivity, and therapies other than topical and systemic steroids. The box shows the current or the worst status during the study periods uveitis inflammation scales based on Standard Uveitis Nomenclature criteria and current BCVA. “Cell” refers to “anterior chamber cells grade”, and “flare” refers to “anterior chamber flare grade”. Case 6 was expressed as OS cell and flare 4 + is error, correct is OS cell and flare are 0. In actually it was too cloudy due to inflammation that the number of cells could not be evaluated. L.P. of Case 6 is error, correct is no L.P., actually she could not recognize the light.” Triangles indicate surgical interventions. Red and blue lines indicate the duration of JIA-U and JIA, respectively. The green line indicates the duration of treatment by a pediatric rheumatologist. ANA: anti-nuclear antibody, RF: rheumatoid factor, BCVA: best corrected visual acuity, F: female M: male, o-JIA: oligo- juvenile idiopathic arthritis, OD: oculus dexter (right eye), OS: oculus sinister (left eye), LP (light perception) ADA: adalimumab, ETN: etanercept, GLM: golimumab, IFX: infliximab, MTX: methotrexate.”

2. The last sentence of the Blindness case 1 (case 6), originally published as:

Although adalimumab (40 mg per 2 weeks) was started, no improvement was seen in the BCVA. Currently, she remains legally blind but continues anti-inflammatory treatment with adalimumab and methotrexate to prevent phthisis bulbi.

Should read:

“Although adalimumab (40 mg per 2 weeks) was started, no improvement was seen in the BCVA. While golimumab was tried to switch, was also no effective (It was not described in Fig. 1 because of very short-term use.) Currently, she remains legally blind but continues anti-inflammatory treatment with adalimumab and methotrexate to prevent phthisis bulbi”.

3. The Authors’ contributions section, originally published as:

SY planned and carried out the patients’ treatment, and drafted the manuscript. MM and HT planned and carried out the patients’ treatment and helped draft the manuscript. AS and YA participated in the patients’ treatment and contributed critical revisions of the manuscript for important intellectual content. TM contributed critical revisions of the manuscript. All authors read and approved the final manuscript.

Should read:

SY planned and carried out the patients’ treatment, and drafted the manuscript. MM planned and carried out all patients’ treatment, and HT treated for some patients, and both revised the manuscript. AS and YA participated in the patients’ treatment and contributed critical revisions of the manuscript for important intellectual content. TM contributed critical revisions of the manuscript. All authors read and approved the final manuscript.

The original article has been corrected.
